# Missing links of melioidosis in India: a cross-sectional analysis of case reports, agrometeorological and socioeconomic factors

**DOI:** 10.1038/s41598-025-27178-4

**Published:** 2025-11-29

**Authors:** Shivvrat Jha, Manaswini Mittal, Laxmi R. Prasad, Somasish Ghosh Dastidar, Sahana Shetty, Damodhara Rao Mailapalli, Pooja Kumari, Harpreet Kaur, Ranita Ghosh Dastidar, Chiranjay Mukhopadhyay, Piyush Behari Lal

**Affiliations:** 1 Center for Emerging and Tropical Diseases, Kasturba Medical College, Manipal, Manipal Academy of Higher Education, Manipal, Karnataka India; 2 Department of Microbiology, Kasturba Medical College, Manipal, Manipal Academy of Higher Education, Manipal, Karnataka India; 3 Kasturba Medical College, Manipal, Manipal Academy of Higher Education, Manipal, Karnataka India; 4 Department of Agricultural and Biosystems Engineering, North Dakota State University, Fargo, USA; 5 Centre of Molecular Neurosciences, Kasturba Medical College, Manipal, Manipal Academy of Higher Education, Manipal, Karnataka India; 6 Department of Endocrinology, Kasturba Medical College, Manipal, Manipal Academy of Higher Education, Manipal,, Karnataka India; 7 Agricultural and Engineering Department, Indian Institute of Technology, Kharagpur, India; 8 Agricultural and Food Engineering Department, Indian Institute of Technology, Kharagpur, India; 9 Division of Communicable Diseases, Indian Council of Medical Research, New Delhi, India; 10 Department of Biochemistry, Kasturba Medical College, Manipal, Manipal Academy of Higher Education, Manipal, Karnataka India; 11 Manipal Institute of Virology, Manipal, Manipal Academy of Higher Education, Manipal, Karnataka India

**Keywords:** Melioidosis, *Burkholderia pseudomallei*, Agrometeorological, Socioeconomic factors, India, South Asia, Bacterial infection, Clinical microbiology, Infectious-disease diagnostics, Policy and public health in microbiology

## Abstract

Melioidosis, an emerging tropical infectious disease and a global threat, lacks a disease prediction model owing to the limited data on case incidences and associated factors. This article focuses on spatial data analysis of melioidosis patients in India, a tropical country considered to be endemic for the disease, to identify gaps in disease reporting. In this study, we screened over 20,000 articles and identified 1,694 patients diagnosed with melioidosis in India between 1953 and 2023. We performed a correlative analysis of patient profiles, case-reporting centres, common misdiagnosed etiologies, susceptible populations, agrometeorological and socioeconomic factors. Our findings suggest that melioidosis can affect individuals of all ages, with a higher prevalence among farmers and individuals with diabetes mellitus, especially adults aged 40–55 years. Most cases are reported during the monsoon season (June to September). Numerous favorable conditions for *Burkholderia pseudomallei* growth exist across India. However, most reported cases are from the south, suggesting under-reporting, under-diagnosis, or misdiagnosis. A “Melioidosis Checklist Index” has been developed as a surveillance tool to enhance case reporting. The melioidosis checklist index combines patient demographics, clinical symptoms, medical and family history, exposure risks, occupation, travel history, and lifestyle factors to assess infection likelihood. Probability scores derived from these parameters provide a structured aid for early diagnosis and case reporting. The study also highlighted the need to enhance regional data collection by raising awareness among vulnerable populations, healthcare providers, and paramedical staff through government initiatives.

**Supplementary Information:**

The online version contains supplementary material available at 10.1038/s41598-025-27178-4.

## Introduction

The imbalance between research needs, research efforts, and government policies stymies the tackling of health challenges. Medical research efforts and policies are often concentrated on limited regional areas that receive greater attention or belong to high-income zones^1^. Diseases emerging from regions that receive little attention pose a significant global health burden and often go unnoticed. One such example is melioidosis, an infectious disease of considerable public health importance in tropical and subtropical regions. It is caused by *Burkholderia pseudomallei*, which is endemic in many countries. It is one of the major diseases to be considered in the differential diagnosis of acute febrile illness^2^.

*B. pseudomallei* is a Gram-negative, motile *β*-proteobacterium, saprophytic bacillus and is classified as a tier 1 select agent as well as a category B biological terrorist by the Centers for Disease Control and Prevention, USA^3^. The organism, first identified in 1911, is shown to infect humans and several animals such as camels, horses, cattle, rodents, dolphins, sheep, pigs, goats, kangaroos, koalas, alpacas, deer, cats, water buffalo, and dogs^4^. It is considered a global threat because it has a mortality rate of up to 50% with antibiotic treatment^5^ and can be aerosolized, which is an important factor for being used as a bioweapon. The bacterium is intrinsically resistant to multiple antibiotics and aseptic solutions through mechanisms that are not completely understood^6^. The infection spreads commonly by inhaling aerosolized bacteria or via abrasion and ingestion of the pathogenic inoculum of bacteria^7^. It mimics pathological symptoms of tuberculosis, Q fever, brucellosis, etc., which interfere with its initial diagnosis^8^. The bacterium is reported in a case study to survive in its host for up to 29 years in a dormant stage^9^. Several other features, such as the lack of robust serological detection^10^ and the requirement of prolonged treatment to avoid relapse^11^, make it difficult to handle a highly dangerous pathogen. Diabetes mellitus (DM) is well-established as a key comorbidity for melioidosis globally^12,13^, and India alone has approximately 101 million people with diabetes^14^. Moreover, melioidosis is more prevalent in rural areas, where basic healthcare facilities are limited and access to diagnosis is poor^15^.

*B. pseudomallei* is a soil-dwelling bacterium whose prevalence in soil depends on weather and soil characteristics such as temperature, rainfall, soil type, soil moisture, and nutrient content^16^. In rural Thailand and northern Australia, increased rainfall has been linked to higher *B. pseudomallei* concentrations in surface soils, which become reservoirs for aerosolized bacteria^17^. India’s diverse climate, with its tropical and subtropical regions, experiences significant rainfall due to the northeast and southwest monsoons^18,19^. In a recent study from Puducherry, farmers were found to be 2.8 times more likely to be seropositive for *B. pseudomallei* compared to non-farmers^20^. Similarly, in Laos, where agriculture is prevalent, melioidosis has notably affected paddy farmers^21^. Soil temperature ranging from 24 °C to 30 °C and moisture content > 40% favor *B. pseudomallei* survival. Sewage dissemination on landscapes, agricultural management practices, and construction work impacts soil conditions (temperature, moisture, and nutrients) and affects bacterial growth^16^. For example, the use of inorganic ammonium, nitrate, and phosphate fertilizers for agricultural and horticultural production promotes bacterial growth in soil^22^. Numerous prior studies have uncovered a battery of environmental and genetic factors that enable the pathogenicity of *B. pseudomallei* strains isolated from different parts of the world. A recent study in southern India (Karnataka) identified *B. pseudomallei* in 1,728 soil samples collected from 20 agricultural sites across the Udupi and Shimoga districts^16^. Hospitals in this region typically report melioidosis cases during the monsoon season (June to September)^23^, a pattern consistent with case reports from other endemic countries^12,24^. A global study predicted the endemicity of melioidosis in 45 countries, 34 of which had not reported any cases until 2016^25,26^. In recent years, most of these 34 countries have published case reports^27^, validating the earlier predictions^26^. While this study and others highlight evidence of melioidosis prevalence in India, the case reporting is limited to a few regions. Overall, estimated cases and fatalities underscore the urgent need for renewed attention from public health officials and policymakers in all endemic countries, including India.

In India, the tropical climate, agricultural management practices (paddy cultivation), frequent flooding, and mineral-rich soil diversity are highly conducive to the growth of *B. pseudomallei*. Despite these favorable factors, no national-level surveillance has been conducted to evaluate the effects of environmental factors on the prevalence and incidence of *B. pseudomallei*. Such a study could enhance the understanding of melioidosis’ geographical distribution and the environmental conditions that support *B. pseudomallei*. Therefore, the objective of this study is to perform a national-level cross-sectional analysis of seven decades of case reports. This aims to improve understanding and provide guidance on managing melioidosis in India and similar tropical and socioeconomic settings. The outcomes include patient profiles, medical centres reporting melioidosis cases, commonly misdiagnosed etiologies, vulnerable populations, and relevant agro-meteorological and socioeconomic factors. This study aligns with the United Nations’ Sustainable Development Goal (SDG) 3 (Target 3.d), SDG 4 (Target 4.7), and SDG 17 (Target 17.6).

## Results

### Publication data indicates the prevalence of melioidosis cases in India

**Fig. 1:**
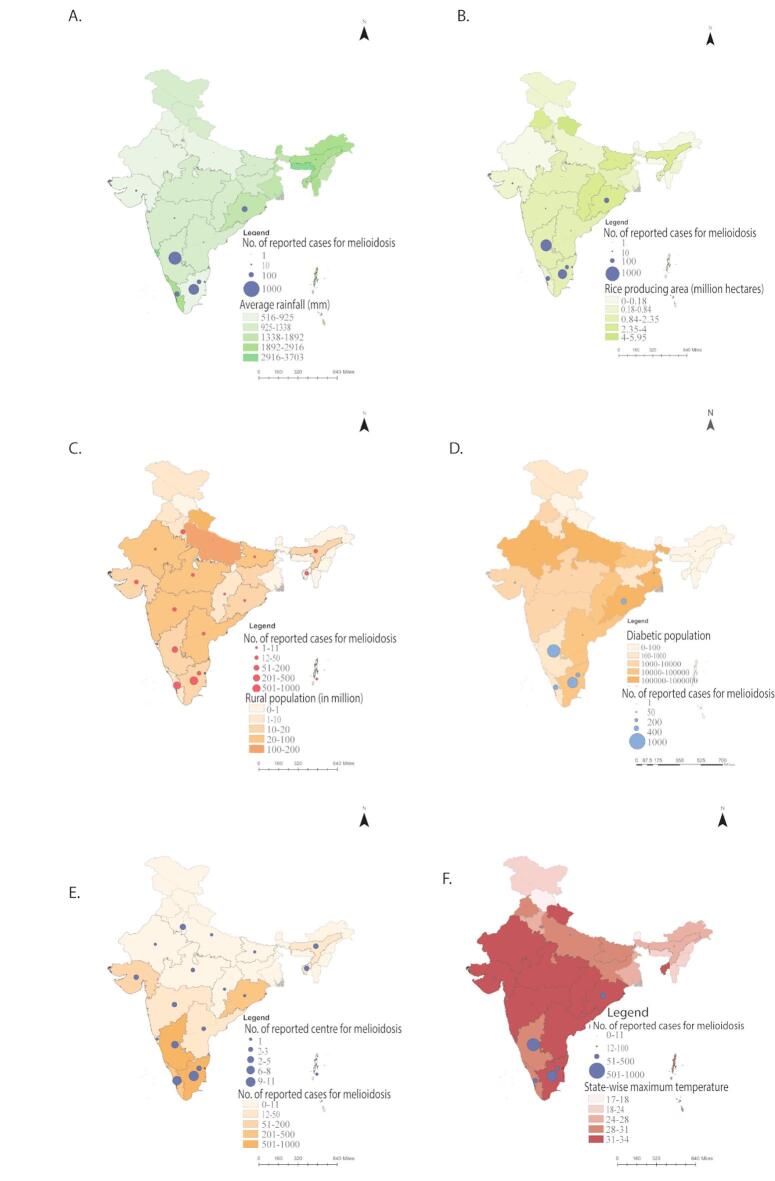
Spatial maps of India for melioidosis case incidences, disease reporting centres, and associated factors. **A**) Spatial map for the average annual rainfall (mm) of 30 years from 1993 to 2022; **B**) Spatial map for the area (million hectares) of rice/paddy field as per Government data 2016; **C**) Spatial map for the rural population (2011 census data); **D**) Spatial map for the predicted diabetes mellitus population; **E**) Spatial map for the number of disease-reporting medical centres, and **F**) Spatial map for the maximum mean air temperature in April. All maps were prepared by feeding the number of case reports and associated factors in the map as per 2011 Government Census data guidelines using ArcGIS Desktop 10.8 software.

 Melioidosis cases were reported across all four regions of India (Fig. S1), but with large variation in numbers (Fig. 1E). There were 1694 case reports of human infection in India between 1953 and 2023 (Table S1). The first case in India was reported in 1953 from central India^33^. However, the subsequent cases were reported from 1991^34^. All cases were diagnosed in India except one where a tourist visited Goa and got diagnosed with melioidosis later in Israel^35^. There were 22 case reports of misdiagnosis with other diseases of similar symptoms and 124 cases of fatal outcomes (Table S1).

### State-wise distribution of melioidosis case reports between 1951and 2023

 Melioidosis cases were reported in 19 states and UTs, mostly in Karnataka (42.6%), Tamil Nadu (33%), Odisha (7.6%), Kerala (7.5%), and Puducherry (4.4%) (Table S3 and Fig. 1A-F). Data lack correlation with coastline or inland regions. Among 13 states and UTs with a coastline (Fig. S3), five had more than 75 cases per state, and the rest had less than 15 cases per state. No cases were reported in Dadra and Nagar Haveli and Daman and Diu and Lakshadweep. In Central India, without a coastline (Fig. S3), five states and UTs had less than five cases per state. No reports of cases from Jharkhand have been published. In the eight states of northeast India without coastline (Fig. S3), melioidosis cases have been reported only in Assam. Similarly, among the states and UTs of northern India without coastline (Fig. S3), only Delhi had seven reported melioidosis cases (Fig. 1A-F).

### State-wise distribution of medical centres diagnosing melioidosis cases

**Fig. 2:**
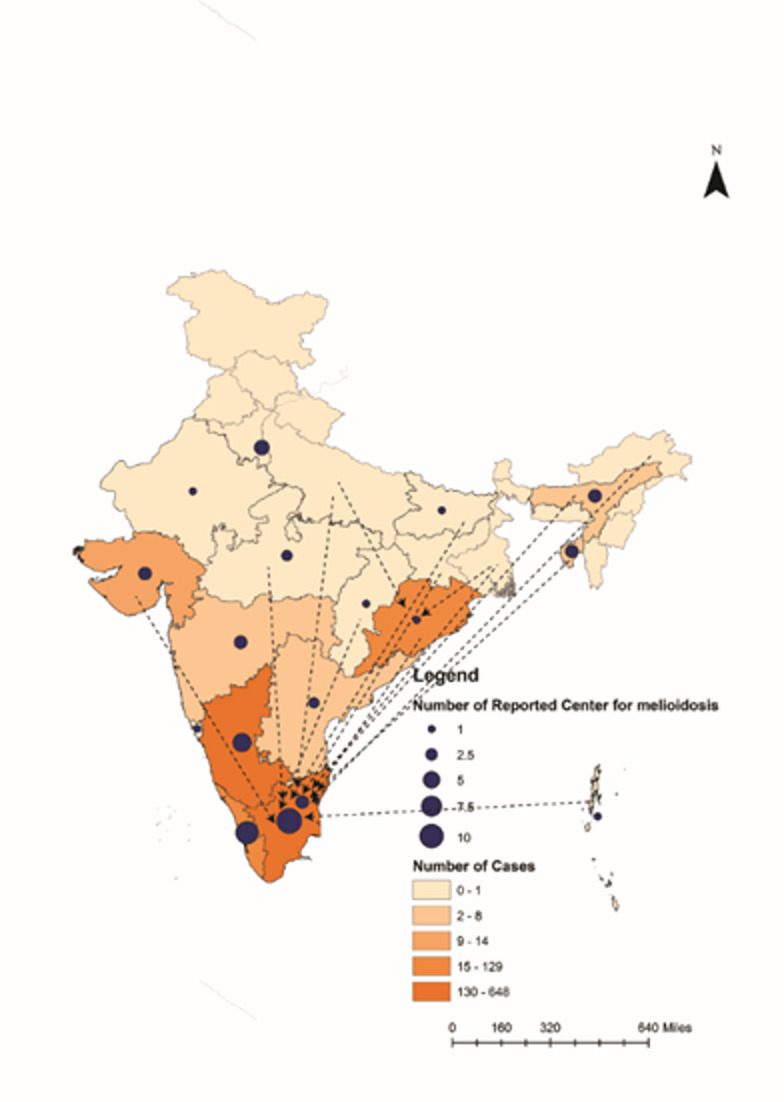
Spatial map showing migration of patients across different states for diagnosis of melioidosis in southern part of India. Dotted line indicates the patient’s movement from one state to another (where arrow shows). Blue circle indicates the number of melioidosis-reporting centres and color gradient shows the number of melioidosis cases. The map was prepared using ArcGIS Desktop 10.8 software.

 There are 73 medical colleges and hospitals (medical centres) in India that have reported melioidosis cases. Most of them are in Tamil Nadu (19.2%), Kerala (15.1%), and Karnataka (9.6%), which constitute 43.8% of the total melioidosis-reporting centres present in the country. Among the 13 states and UTs that share a coastline, Tamil Nadu, Kerala, and Karnataka had more than five melioidosis-reporting medical centres. All seven states of central India without coastline had less than seven melioidosis-reporting medical centres. Among the eight states of northeast India without coastline, only Assam had three melioidosis-reporting medical centres (Fig. 1C and Table S8). Similarly, none of the medical centres in the states and UTs without coastline in northern India have reported cases, except Delhi (four centres). In some cases, patients traveled to medical centres in other states for better health checkups (Fig. 2 and Table S12).

### Distribution of melioidosis case reports against population density

**Fig. 3:**
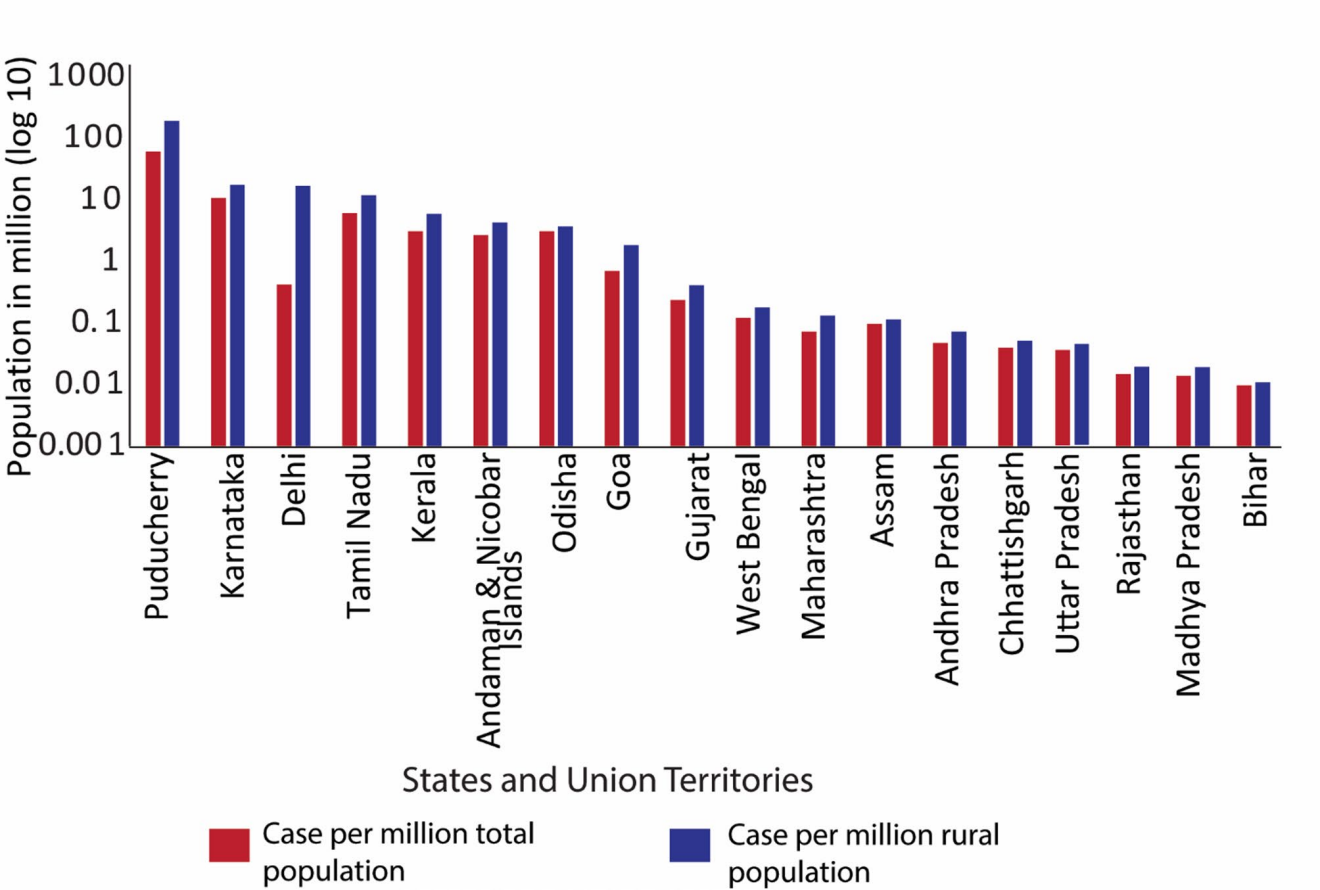
Melioidosis Incidence rates in different states of India. Red bars indicate the number of cases per million population of the state and blue bars indicate the number of cases per million rural population of the state. For incidence rate, the total number of case reports (1953–2023) was normalized against the population of the states obtained from 2011 Government census data.

 A higher number of cases are expected in densely populated regions (Fig. 3 and Table S6); however, no correlation was observed between case numbers and population size. Among the eight most populated states (> 50 million) (Table S6), only Tamil Nadu and Karnataka reported > 400 cases; others had < 12 cases (Table S6). Uttar Pradesh, despite a population of 200 million, had very few cases. In eleven of the medium-populated states, only Odisha and Kerala had > 100 cases; others had < 7 cases. Among 15 of the less populated states/UTs, only Puducherry reported a notable 75 cases while the rest of the states had zero or less than four reported cases. Analysis by rural population (Fig. 1C and Table S6) showed that Odisha with 129 cases was the only high-rural state (> 75% rural) with significant incidence while others had either zero or single reported cases. In states with 50–75% rural population, Karnataka (648), Tamil Nadu (434), and Kerala (102) had high case counts while the rest had either zero or fewer than 12 cases. Overall, Puducherry, Karnataka, Tamil Nadu, Odisha, and Kerala had incidence rates of 2–60 cases per million (Fig. 3 and Table S6). In other regions, rates were below 0.1 per million, suggesting low reporting regardless of population density. Incidence per million was calculated by dividing the total cases in a state or UT by its population.

### Distribution of melioidosis case reports against gender, age, and occupation of the patients

**Fig. 4:**
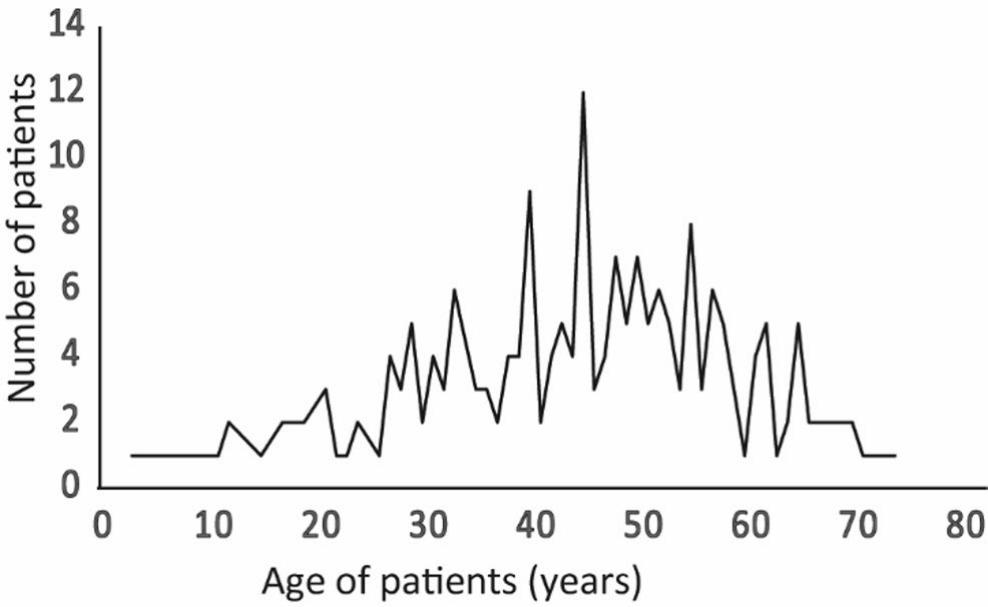
Age wise distribution of reported Indian melioidosis patients from 1953 until 2023. The age of 205 patients, mentioned in the publications, were plotted as a line graph.

**Fig. 5:**
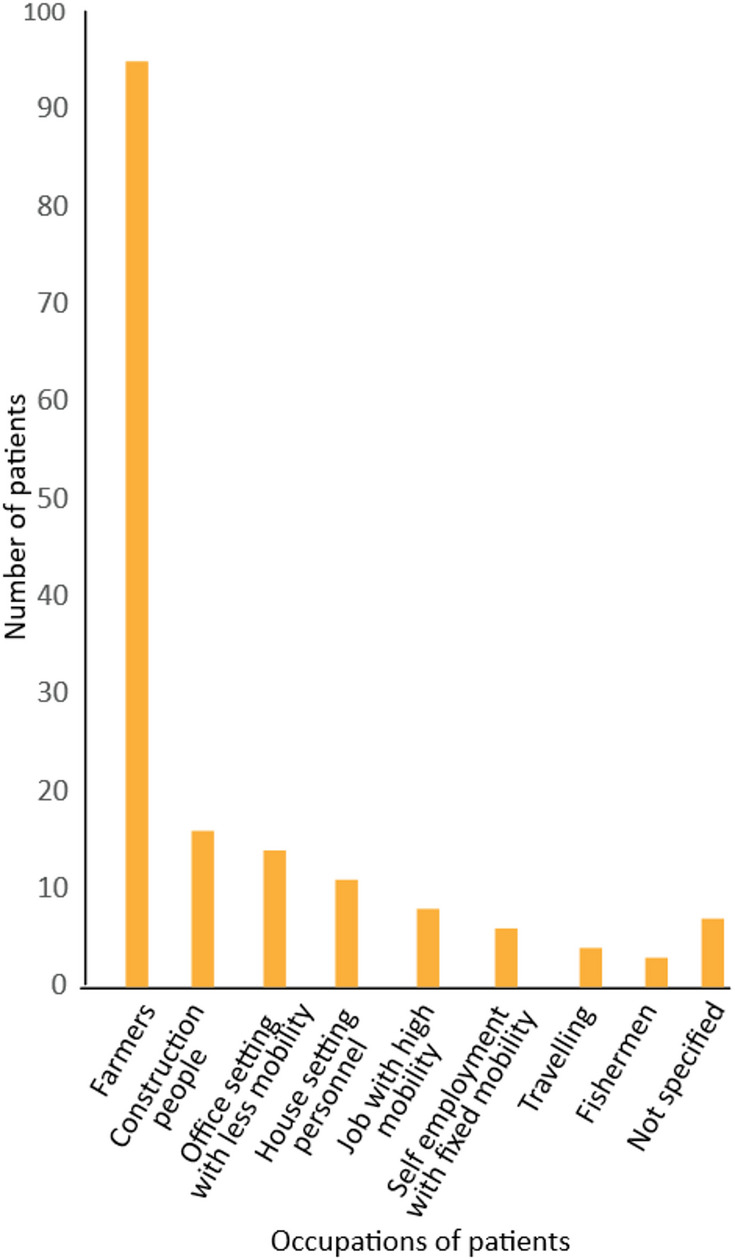
Occupation of Indian melioidosis patients from 1953–2023. Data were obtained from the published case reports (*n* = 164 cases) and were categorized into nine groups based on their nature of job, mobility, exposure to soil and water.

Among 1694 case reports, 1459 case reports contained gender information that included 1155 (79.2%) male patients and 304 (20.8%) female patients (Table S1). 205 reports contained the age of patients that varied from 35 weeks to 78 years (Fig. 4; Table S1). The median age of patients was 45 years, and most of the patients were between 40 and 55 years. 164 reports contained occupation information that indicated farmers are most susceptible to the disease (Fig. 5).

### Distribution of melioidosis case reports against diabetes and other risk factors

**Fig. 6:**
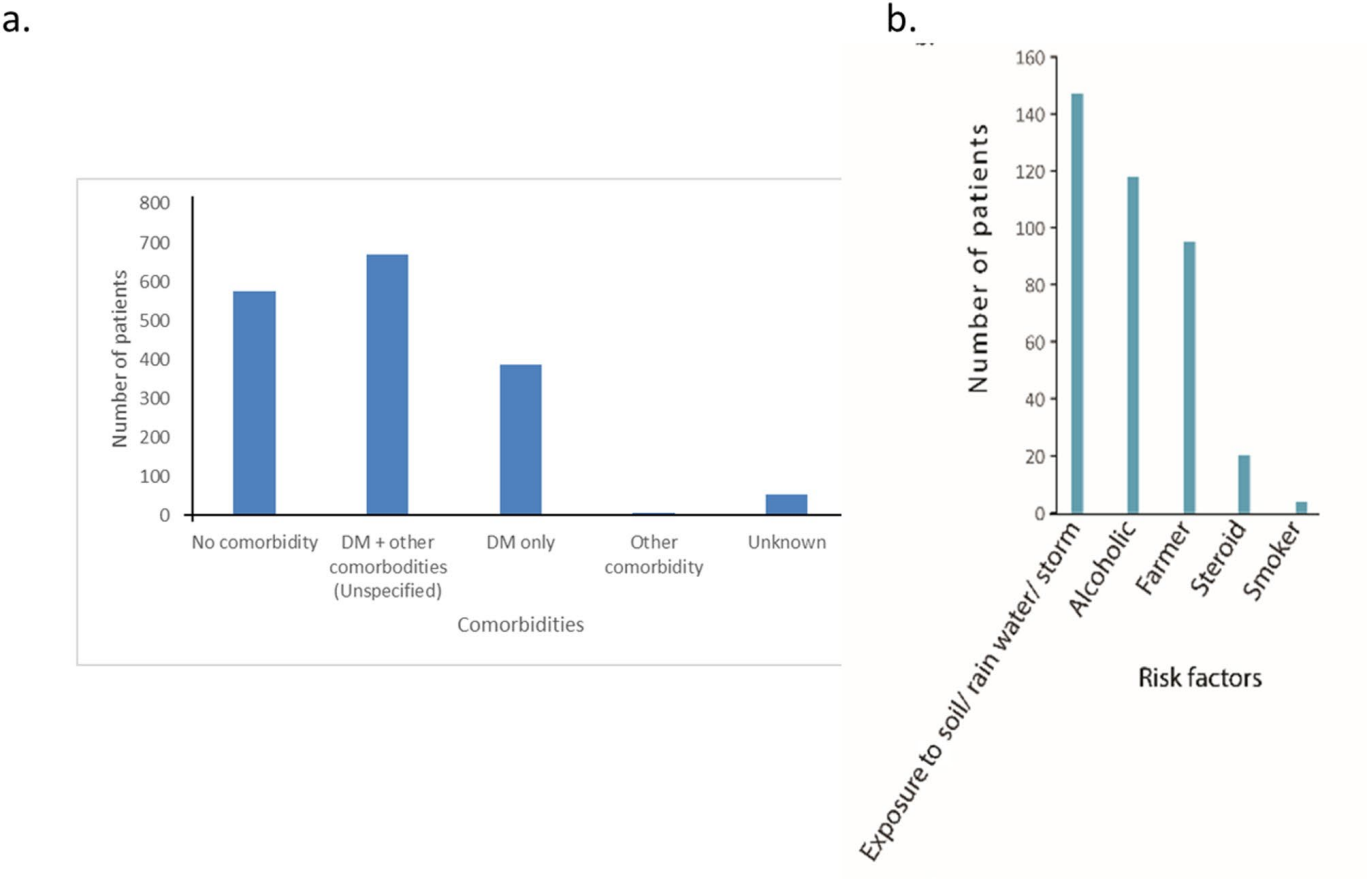
Comorbidities and risk factors of melioidosis patients mentioned in case reports from 1953 to 2023. **a**) The bar graph indicates the number of patients with no comorbidity, diabetes mellitus (DM) as a major comorbidity, and other comorbidities (as mentioned in table S2) that may be associated with the disease. “Unknown” indicates the number of patients with unclear comorbidity information. **b**) The bar graph indicates the number of patients with major risk factors that may be associated with melioidosis. Exposure to soil/rainwater/storm indicates that the patient was exposed to either of the mentioned conditions.

 Among 1694 case reports, 1056 case reports contained comorbidity. A total of 387 patients were reported with DM alone, and others were reported with single or multiple comorbidities with or without DM (Fig. 6a, Table S1, and Table S2). Sixty-two diabetic patients were diagnosed with acute kidney injury (AKI), which may be the outcome of melioidosis infection stress. Rare presentations, such as brain, spine, and muscle involvement, and various rare combinations, were also reported in some cases. 147 case reports indicated additional risk factors for the disease, such as people with exposure to soil and rain and alcohol addiction (Fig. 6b).

 There was no correlation between total incidences and the predicted diabetic population (Fig. 1D and Table S7). For example, none of the three states (Uttar Pradesh, Bihar, and Andhra Pradesh) with the most diabetic patients (> 10 M patients) had a significant number of reported cases. Among 15 states and UTs with one to ten million diabetic patients, Karnataka (648 cases) and Tamil Nadu (434 cases) had significant reported cases; the rest had zero or less than 12 reported cases. Similarly, among 17 states and UTs with less than one million diabetic patients, Kerala (102 cases) and Puducherry (75 cases) had significant reported cases; the rest had zero or less than four reported cases.

### Distribution of melioidosis case reports against rainfall in States and UTs

 There are 15 states and UTs that get more than 1500 mm of average annual rainfall from 1993 to 2022 (Fig. 1A; Table S3). However, Kerala was the only state in this group that had 102 melioidosis-reported cases; the rest had either zero or less than 12 melioidosis-reported cases. Among 12 states and UTs with average annual rainfall (Table S3), only Karnataka (648 cases), Odisha (129), and Puducherry (75 cases) had significant reported cases; the rest had either zero or less than nine reported cases. The coastal cities of Karnataka and Maharashtra receive more rain than the inland area of the state (Fig. S3; Table S4). Differential case report data for the two regions are not available. Among eight states and UTs with low annual rainfall (Table S3), Tamil Nadu (434 cases) had significant reported cases; the rest had either zero or less than five reported cases.

### Distribution of melioidosis case reports against temperature in States and UTs

 A correlation between case and temperature could not be made, as only 6 among 22 states (Odisha, Karnataka, Tamil Nadu, Kerala, Tamil Nadu, and Puducherry) with the highest mean temperature above 35 °C had reported more than 100 cases. None of the 14 states with the highest mean temperature in the range of 22 °C to 30 °C reported more than five cases (Fig. 1f and Table S10).

### Distribution of melioidosis case reports against paddy field areas in States and UTs

 There were nine states with an average paddy growing area of more than two million hectares; Odisha was the only state in this group that had 129 melioidosis cases; the rest of the states had either zero or less than five melioidosis cases reported. Seven states had an average paddy growing area of one to two million hectares; Tamil Nadu and Karnataka were the only states in this group that had more than 400 cases reported; the rest had either nil or less than five cases reported. 20 states had an average paddy growing area of less than one million hectares; Kerala and Puducherry were the only states in this group that had more than 75 melioidosis cases reported; the rest had either nil or less than 15 melioidosis cases reported (Fig. 1b; Table S5).

Uttar Pradesh had a maximum paddy growing area; however, there are very few melioidosis cases reported so far. The other central inland states had an insignificant number of cases (Fig. 1B; Table S5). Northern states contain a variable range of rice-growing fields. However, none of the Northern states had melioidosis cases, except in Delhi (7 cases). Northeast states contain less than one million hectares of rice fields except Assam (2.35 million hectares) (Fig. 2b; Table S6).

### Distribution of soil minerals across India that May favor bacterial growth

 We created a thorough analysis chart of soil minerals, such as low salinity, low content of nitrogen, manganese, iron, phosphorus, potassium, zinc, and organic carbon, and neutral pH, against melioidosis cases in all states (Table S9). However, there were not enough case reports to determine the correlation between bacterial prevalence and the mineral content in the soil. All data on soil mineral composition are provided in Table S9 for reference in future case reporting and analysis.

### Analysis of unpublished case reports in Udupi district to create a disease prediction model

**Fig. 7:**
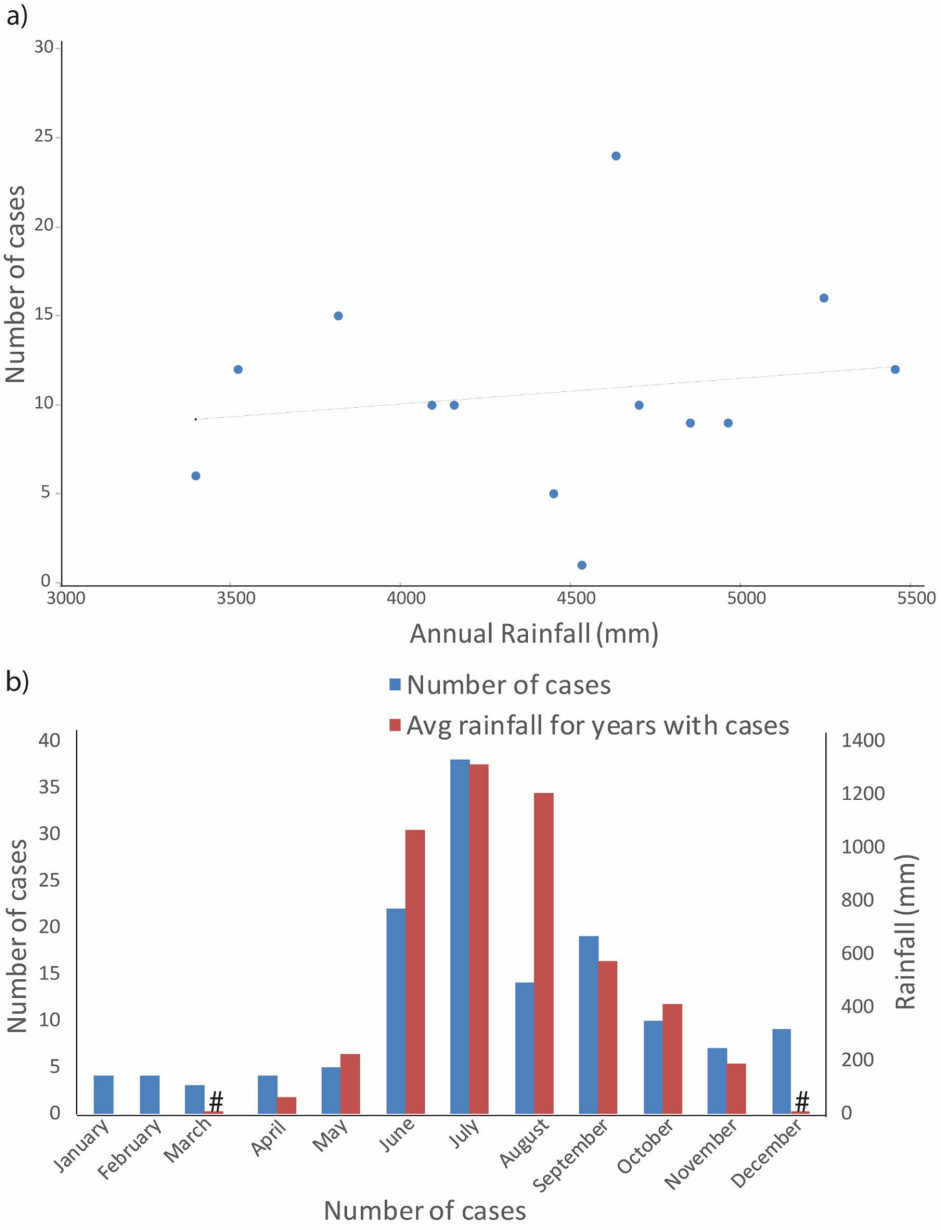
Melioidosis cases reported at Kasturba Hospital from 2010 to 2023 and rainfall data of Udupi district. **a**) A scatter plot displaying yearly case reports in relation to precipitation levels (R² = 0.0262); **b**) A bar graph illustrating the relationship between the number of cases per month (blue bars) and the average rainfall in millimeters (red bars). A “hash” symbol denotes instances of relatively negligible values.

 To generate a regression correlation model of cases against precipitation, we analyzed monthly precipitation against the 139 cases at Udupi station of IMD, Pune, between 2010 and 2023. Although 66.9% of cases occurred during the monsoon season (June to September) (Fig. 7b), we could not establish a significant correlation between annual cases and average annual precipitation (in mm). This lack of pattern is likely due to the relatively small number of cases available, which limited the statistical power of the analysis (Fig. 7a; Table S11). Nonetheless, we observed that the number of male patients was 3.1 times higher than female patients, with median ages of 50.5 and 56 years, respectively.

## Discussion

Melioidosis, an often-neglected tropical disease, remains underdiagnosed and misdiagnosed worldwide, including in India. The first documented case in India was reported in 1953, involving a Scottish coal miner in Bihar, but the disease was only identified posthumously^33^. It wasn’t until 1991 that a second case was reported by Indian scientists in Mumbai^34^. Despite its high fatality rate, which is greater than many tropical infections, melioidosis lacks standardized diagnostic guidelines and protocols in India^28,36^. This work aims to address this gap by compiling a comprehensive dataset of melioidosis cases across India.

### Diagnosis of melioidosis is centered around a handful of hospitals in India

Recent literature highlights several southern Indian regions as “hotspots” for melioidosis^28,37^, with numerous cases reported from these areas. In our study, we identified melioidosis cases from across India, including the northern, northeastern, and central regions. We found that diagnosis is predominantly concentrated in a few hospitals, primarily in southern India. Out of 8,673 primary and district health care centres across the country^38,39^, only 73 have reported melioidosis cases, with 96% of reports coming from just 30 centres in five states: Karnataka, Tamil Nadu, Kerala, Puducherry, and Odisha (Table S8).

Coastal cities with comparable economic development status and similar climatic zones are highly likely to have exposure to a similar group of endemic infectious diseases^40^. However, despite India’s extensive coastline across 14 states and UTs, cases are reported mainly from a handful of centres in five coastal states of the country. This suggests a pattern of underreporting, underdiagnosis, or misdiagnosis with other diseases due to a lack of awareness at different levels. Recent reports from northern, central, and northeastern India indicate that melioidosis might be more widespread in India than currently recognized.

### A lack of robust data for determining correlation between densely populated States and incidences

India, with its projected population of 1.4 billion, exhibits significant regional disparities in disease reporting. Densely populated states are predicted to have a higher incidence rate^41^. Despite the high population density, melioidosis cases are predominantly reported from a few centres in southern states, with very few cases reported in Uttar Pradesh, the most populous state, until 2023 (Table S6). This uneven distribution suggests that current case data does not accurately reflect the true incidence across the country. For instance, states with high populations like Karnataka, Tamil Nadu, Kerala, and Odisha report fewer cases per million people (less than 10 cases per million people) compared to less populous Puducherry (60 cases per million people) (Table S6). This discrepancy indicates that the actual incidence of melioidosis might be higher than current reports suggest, highlighting the need for improved diagnostic and reporting efforts, especially in densely populated regions.

Moreover, melioidosis is more prevalent in rural areas, where basic healthcare facilities are limited and access to diagnosis is poor^15^. In India, over 68% of the population resides in rural areas (Table S6), yet about 75% of healthcare resources are concentrated in urban centres. This disparity means that rural populations are particularly vulnerable to melioidosis. There is a lack of seroprevalence data and case reports from these areas. This underscores the urgent need for comprehensive geographical mapping and improved diagnostic infrastructure to address melioidosis across urban and rural settings.

### DM and hypertension are the major comorbidities that put several States in a high-risk zone

Diabetes mellitus (DM) is well-established as a key comorbidity for melioidosis globally^12,13^. Our data further reveals a significant association between melioidosis and other conditions such as hypertension and chronic kidney disease, with DM being the primary comorbidity. Besides DM, other comorbidities such as liver disease, malignancy, lung disease, coronary artery disease, human immunodeficiency virus, and renal diseases were reported in about 5% of cases (Fig. 6a, Table S1 and Table S2). In India, where approximately 101 million people have diabetes (Fig S2)^14^, primarily in states like Uttar Pradesh, Bihar, Andhra Pradesh, and West Bengal, the number of reported melioidosis cases remains low (Table S7). This discrepancy suggests a potentially high burden of melioidosis in diabetic populations if better diagnosed. Furthermore, a significant proportion of diabetic patients remain untreated, indicating that the true incidence of diabetes and melioidosis may be underestimated. Besides DM, patients were reported with other risk factors that are associated with several melioidosis cases^12^, such as excessive alcohol use and chronic lung disease, for which our case report dataset is not robust. Most case reports lacked the vital information of the patient’s comorbidity and associated risk factors, required for generating a prediction model. Future case reports should comprehensively document all risk factors to improve predictive models and diagnosis.

### Farmers, rural populations, and mostly 40 to 55-year-old men are reported with higher susceptibility to melioidosis

Subsistence agriculture remains a primary occupation for 70% of rural households in India, with 82% being small and marginal farmers^42^. Our data indicates that farmers are particularly vulnerable to melioidosis (Fig. 5), which aligns with global findings highlighting agriculture as a significant risk factor^13^. In a recent study from Puducherry, farmers were found to be 2.8 times more likely to be seropositive for *B. pseudomallei* compared to non-farmers^20^. Similarly, in Laos, where agriculture is prevalent, melioidosis has notably affected paddy farmers^21^. The median age of melioidosis patients in our dataset was 45 years, with a peak incidence between ages 40 and 60 years (Fig. 4), consistent with other studies^13^. Given that over 27% of India’s population is over 40 years old^29,43^, many cases likely remain unreported, emphasizing the need for improved diagnosis and reporting. Our findings from the single centre case reports at Kasturba Hospital, Manipal in the Udupi region between 2010 and 2023 (Table S11) also show that males are four times more likely to contract melioidosis than females, which corroborates global data^37,44^. This disparity may be linked to occupational exposure, as females, despite participating in agriculture, might have different exposure patterns. Further research with detailed gender and occupational data is needed to fully understand these dynamics and enhance disease management strategies.

### People of States with paddy farming land are prone to melioidosis infection

States with extensive paddy farming area are expected to have higher bacterial prevalence due to the unique soil environments in wetlands^45^, which support the growth of *B. pseudomallei*^46,47^. These soils, with their oxic surface, anoxic bulk, and rhizosphere layers, provide an ideal habitat for this bacterium^45,48^. Our data reveals that farmers, particularly those working with wetland crops like paddy, sugarcane, and jute, are highly susceptible to melioidosis (Fig. 5). Despite this, reported cases from major paddy-growing states in central India (Fig S1) are disproportionately low compared to the expected incidence (Table S5). Enhanced case reporting and surveillance in these high-risk areas are crucial for a more accurate assessment of bacterial prevalence.

### People in States with high rainfall are prone to melioidosis infection

*B. pseudomallei* is believed to become aerosolized during activities like plowing and heavy rainfall, leading to inhalational melioidosis^49,50^. In rural Thailand and northern Australia, increased rainfall has been linked to higher *B. pseudomallei* concentrations in surface soils, which become reservoirs for aerosolized bacteria^17^. India’s diverse climate, with its tropical and subtropical regions, experiences significant rainfall due to the northeast and southwest monsoons^18,19^. Despite this, reported melioidosis cases are notably low in high-rainfall states, except for Kerala, Puducherry, and Karnataka (Table S3). India receives different levels of rainfall across the country from the northeast monsoon and the southwest monsoon^18^. For example, an analysis of rainfall in coastal and inland parts of states-with-coastline indicates high rainfall in the coastal areas, especially in west coast regions, and less rain in the inland regions (Fig S3 and Table S4)^13^. It indicates that the coastal regions of the Western Ghats with high rainfall are in a greater risk zone for melioidosis incidences than the regions with less rainfall^13^. Conversely, states like Rajasthan and northern regions, with lower rainfall, may face different risk factors (Table S3). Enhanced case reporting across all states is essential to better understand the relationship between rainfall patterns and melioidosis incidence in India.

### Tropical climate and suitable soil properties constitute important risk factors for *B. pseudomallei* in several States

Environmental exposure in tropical regions is a well-documented risk factor for melioidosis, with *B. pseudomallei* thriving in such climates^31,32^. India’s diverse climate, predominantly tropical with seasonal wet and dry periods, creates ideal conditions for this bacterium^47,51^. The average mean air temperature across India, ranging from 25 °C to 35 °C (Table S10)^52–54^, is favorable for *B. pseudomallei* growth^55^. It is not known how bacteria survive in that hostile temperature. One explanation is that bacteria may survive intense heat by residing deeper in the soil, resurfacing during wet seasons when groundwater levels rise^56^, thereby increasing the risk of aerosolized bacterial inhalation^57^. Interaction with moist soil, water bodies, and flood-prone areas has been frequently observed in melioidosis cases^58^. Soil conditions, such as low concentrations of manganese, nitrogen, cadmium, phosphorus, potassium, calcium, and magnesium, influence bacterial growth, although the role of iron remains debated^16,32,46^. Soil types also affect bacterial survival; clay soils, typically more anaerobic, and sandy soils, which are more aerobic, present different growth conditions for *B. pseudomallei*^32^. Soil pH plays a crucial role, with acidic soils often providing a competitive advantage for *B. pseudomallei*. Neutral soil pH provides maximum diversity of bacteria in soil. Unlike most bacteria, *Burkholderia sp.* has a competitive advantage in acidic soils, which helps them outcompete several microbes^47^. Nitrates, urea, and phosphate increase *B. pseudomallei* load in sand^22^. Given these factors, Indian regions with extensive paddy cultivation fields and anoxic soil environments could be significant reservoirs for *B. pseudomallei*. Preliminary studies have identified the presence of *B. pseudomallei* in coastal soils of India^59^, but more comprehensive soil surveys are needed to assess its distribution nationwide. A national soil survey and controlled laboratory growth analyses under various conditions will provide valuable insights into the bacterial prevalence and its potential impact on melioidosis incidence. This data will be crucial for improving disease prediction and management strategies in India.

### Misdiagnosis, lack of awareness, and negligence are the major challenges

Melioidosis diagnosis relies on isolating *B. pseudomallei* from blood, urine, sputum, skin lesions, or abscesses, and increasingly through a series of biochemical tests, culture on selective medium (Ashdown’s agar), advanced PCR and MALDI-TOF assays^10,13,57,58^. Despite these methods, the disease remains significantly underdiagnosed, primarily due to the lack of diagnostic facilities (Biosafety Level 2 laboratory) in low-income rural areas and limited awareness among healthcare professionals^60^. Due to the similarity in disease etiology between melioidosis and infections caused by *Mycobacterium tuberculosis*, *Pseudomonas aeruginosa*, and *Burkholderia cepacia*, melioidosis cases are often misdiagnosed as these other infections (Table S1). In India, it took 38 years from the first reported case in 1953 to identify a second case in 1991 due to these gaps^33,34^. Despite increased awareness in recent years, melioidosis remains underreported nationwide, highlighting the critical need for stronger government initiatives to improve surveillance and reporting.

### Need for checklist index: A tool for melioidosis surveillance

 Our analysis reveals that melioidosis cases are detected primarily in a few specialized centres, often after patients from regions with limited diagnostic resources are referred for further investigation. For example, patients from West Bengal, Jharkhand, and Assam are often diagnosed in Odisha and Tamil Nadu, indicating a regional disparity in diagnostic capabilities (Fig. 2 and Table S11). Misdiagnosis and delayed reporting contribute to the high fatality rate, with 28 cases of misdiagnosis and 120 deaths recorded in our dataset (Table S1), though these numbers may be underreported due to inadequate follow-up. Furthermore, nearly all case reports lacked critical information necessary for developing a robust correlation model. To address these issues, we propose a “melioidosis checklist index” (Table S13) to be used at all levels of healthcare, from primary to tertiary care centres. The melioidosis checklist index incorporates patient demographics, clinical symptoms, medical and family history, exposure risks, occupation, travel history, and lifestyle factors to estimate the likelihood of infection. Probability scores generated from these parameters provide a systematic tool to support early diagnosis and standardized case reporting. The probability factors presented in Table S13 (b) are derived from favorable determinants for melioidosis and *B. pseudomallei* growth, including climate, soil type, rainfall, and reported case incidences in each state, rather than a numerical scoring system. These factors highlight regions with a higher likelihood of infection and serve as a structured tool to support early diagnosis and systematic case reporting. This checklist is designed to address the persistent issue of underreporting and to support government-led efforts in strengthening national surveillance across India. By integrating population and environmental factors through a One Health approach, it serves as a vital tool for educating clinical, pre-clinical, and paramedical staff, and the general public, about melioidosis nationwide. This “Melioidosis Checklist Index” is also being circulated and implemented at the centres participating in Indian Council of Medical Research’s (ICMR’s) Mission- Task Force for a multicentre case reporting study on melioidosis to enhance diagnosis across Indian states. Improved case reporting, enhanced diagnostic facilities, and better follow-up procedures are crucial for accurately understanding and managing melioidosis in India. The government should adopt this framework for policymaking and nationwide awareness programs.

## Limitations of this study

In this work, we analyzed published cases of melioidosis across India to explore potential correlations with agro-meteorological and socio-economic factors. However, several limitations restricted the development of a robust mathematical model. Key challenges included insufficient case reports from many states and incomplete documentation of associated variables such as gender and occupation. Our data also indicated lower mortality rates in some states compared to global estimates, likely due to suboptimal case follow-up. These findings highlight the need for comprehensive reporting of all disease-associated factors and more detailed analyses of linked determinants. Notably, significant epidemiological insights unique to India have emerged mainly in the past two decades, primarily from KMC Manipal and other centres in Southeast India (Table S1). Moving forward, broader nationwide epidemiological reporting, alongside agro-meteorological and socio-economic assessments, is essential. This article and the proposed ‘checklist’ serve as a foundation for such future efforts.

## Conclusions

A melioidosis checklist index, identifying potential risk factors for the disease, was developed in this article to help primary care clinicians improve diagnosis and reporting. The study recommends incorporating melioidosis as a legally notifiable disease in Indian health policy and providing clinicians and microbiologists with proper training, education, and guidelines for its recognition and management. It is imperative that the government adopt this initiative as a foundation for policymaking and nationwide awareness programs.

Due to the absence of a vaccine or highly effective antibiotic therapy, preventive measures remain the most effective strategy for controlling melioidosis. Individuals with diabetes and paddy farmers in frequent-rainfall areas are advised to use protective equipment, such as boots and gloves, and to cover open wounds, cuts, or burns to avoid direct contact with saturated soil and water. India has highly favorable conditions for melioidosis, suggesting that the actual number of cases may exceed current reporting. Future efforts should focus on refining the melioidosis checklist, improving patient recruitment procedures, and enhancing reporting systems across various levels of healthcare.

## Methods

### Literature search and melioidosis case reports

**Fig. 8:**
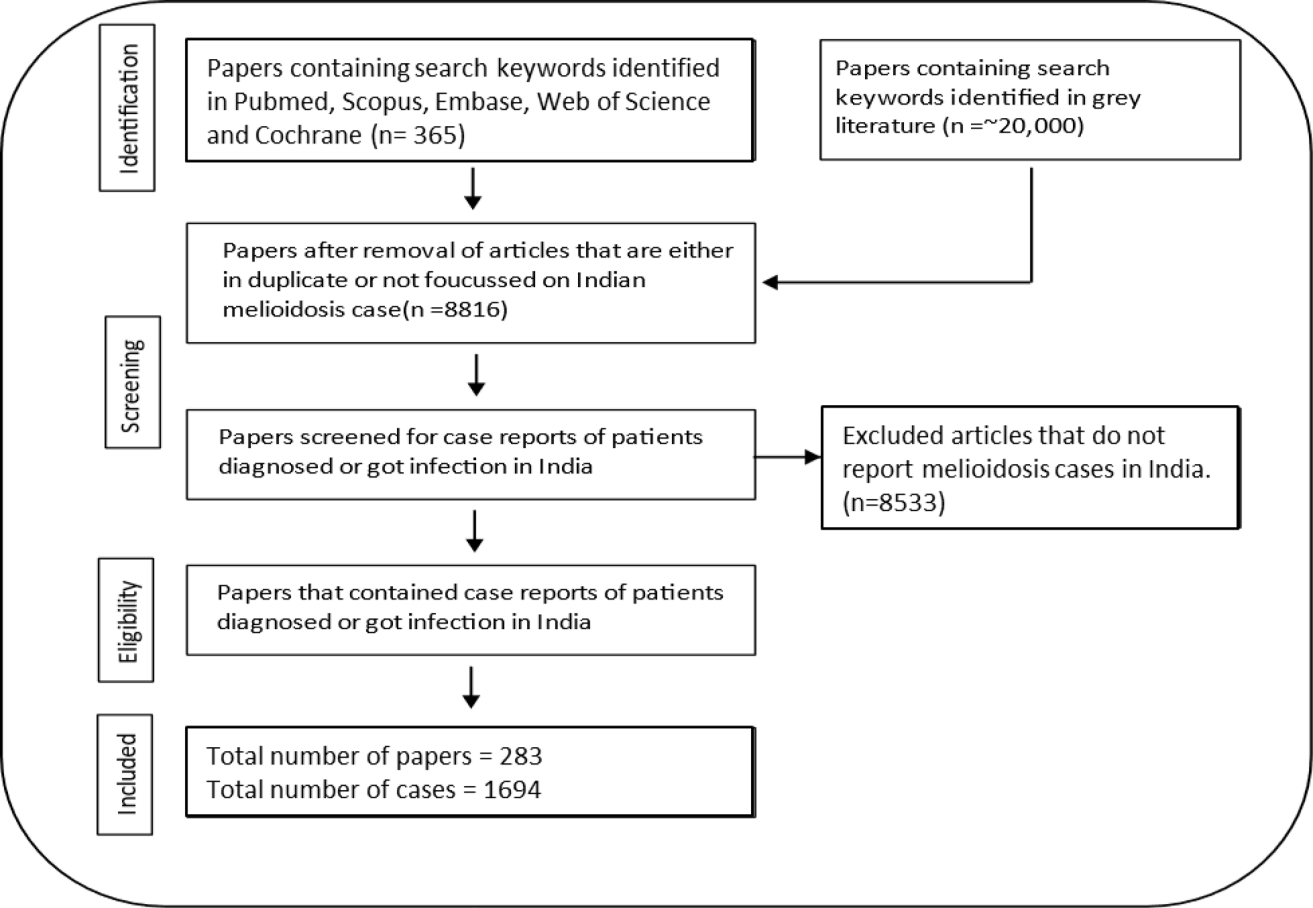
Schematic representation of the methodology to identify case reports published in India. Case reports of melioidosis patients, infected or diagnosed with the disease in India, were identified in the databases PubMed, Scopus, Web of Science, Cochrane, Embase, and grey literature (Google Scholar) from 1953 to 2023.

Melioidosis case reports published in India were from online databases and grey literature (Fig. 8), using the keywords “*Burkholderia pseudomallei”*, “Melioidosis”, and “India”. Each case report and the article were screened for the patient’s clinical representations, lifestyle, diagnosis centre, and location (Tables 1 and Table S1). To maintain uniformity across studies in which patient addresses were frequently unavailable and contributing authors represented multiple regions, the first author’s institutional affiliation was used as a proxy for patient location. We also carried out a single-centric retrospective study for the Udupi district in Karnataka, India. Anonymized patient data, including district, age, gender, and the month and year of melioidosis diagnosis, were used for correlation analysis. Data was obtained from the medical archives with permission from the Medical Superintendent, Kasturba Hospital, Manipal. Due to the retrospective nature of the study, the Institutional Ethics Committee of Kasturba Medical College, Manipal and Kasturba Hospital, Manipal, India, waived the requirement for obtaining informed consent. The waiver was granted as no biological or patient samples were used, and all data were fully anonymized. All methods were conducted in accordance with the relevant guidelines and regulations. Ethical clearance (IEC number: 308/2022) was obtained from the Institutional Ethics Committee of Kasturba Medical College, Manipal and Kasturba Hospital, Manipal, India.

**Table 1:** Resources for the materials and data collected in this study.

S. Number	Type of data	Timeline (Year)	Resources
1	Melioidosis case reports	January 1912-December 2023	PubMed, Scopus, Web of Science, Cochrane, Embase, and grey literature.
2	Total, rural and urban population	2011	Government census 2011
3	Diabetic population data	2021	National Health Registry (cbhidghs)
4	Annual rainfall data	1993–2022	India Meteorological Department, Pune
5	Paddy area	2021	Indian Ministry of Agriculture
6	Soil properties	2022	Soil Health Card, Ministry of Agriculture and Farmers Welfare

### Census and diabetic prevalence

Population data was obtained from the most recent census (2011) of Government of India public database^21^. In this cohort study, states and UTs are grouped into three categories (Table S6; abbreviations as per Fig S1). Data for the cases from 1991 to 2023 are normalized based on the 2011 census data, as it is the midpoint for this cohort study tenure. Diabetes mellitus (DM) (2021 to 2022) information was obtained from the National Health Profile 2022, 17th issue. The diabetic percentage (2021) from the non- communicable diseases (NCDs) clinic was used to predict a value for the total diabetic population in a state or Union Territory (UT).

### Paddy growing area

Information on state-wise paddy area was collected from published reports of India Meteorological Department (IMD), and the Department of Agriculture and Farmers Welfare of the Government of India in 2016. States and union territories were categorized into three groups based on average paddy-growing area (Table S5).

### Soil characteristics

Information on regional soil properties and nutrients that promote growth of *B. pseudomallei* is obtained from the Government of India, Department of Agriculture and Farmers Welfare and Ministry of Agriculture and Farmers Welfare^12,22–24,30^ (Table S9).

### Precipitation

State-wise annual precipitation data from 1993 to 2022 were obtained from IMD, and they were grouped into high, medium, and low rainfall (Table S3 and Fig S1). Annual precipitation criteria are listed in table S3, and groupings of states and coastline categorizations are shown in Fig S1.

### Air temperatures

The monthly mean temperatures of Indian States and UTs were obtained from the IMD, and the Oxford School Atlas 36th edition was used for generating spatial maps of India (Fig. 1F and Table S10). Based on mean April temperatures, all states and UTs were grouped into marginal, medium, and hottest categories. For determining melioidosis prevalence in states with elevated temperature, the number of case reports in each state was analyzed.

### Unpublished case reports from Udupi to create a disease prediction model

Melioidosis cases reported between 2010 and 2023 in Karnataka state’s Udupi district were used to develop a disease prediction model. This information was obtained from Kasturba Hospital, Manipal (Table S11).

### Data analysis

The data analysis of annual precipitation, melioidosis case reports, and disease-associated factors was done using Microsoft Access 2010. The spatial maps were created using ArcGIS Desktop 10.8 software. Descriptive and regression analyses were performed using Microsoft Excel 2010.

## Supplementary Information

Below is the link to the electronic supplementary material.

Supplementary Material 1

Supplementary Material 2

Supplementary Material 3

Supplementary Material 4

Supplementary Material 5

Supplementary Material 6

Supplementary Material 7

Supplementary Material 8

Supplementary Material 9

Supplementary Material 10

Supplementary Material 11

Supplementary Material 12

Supplementary Material 13

Supplementary Material 14

Supplementary Material 15

## Data Availability

All datasets generated in this study are included in the manuscript or supplementary files.
