# Supplementary material for: Missing links of melioidosis in India: a cross-sectional analysis of case reports, agrometeorological and socioeconomic factors
Source: Sci Rep. 2025 Nov 29;15:43237. doi: 10.1038/s41598-025-27178-4 (PMC12680645; doi:10.1038/s41598-025-27178-4)
Supplement: Supplementary file 1 — Supplementary Material 1 [file 41598_2025_27178_MOESM1_ESM.docx]

**Title:** Missing links of melioidosis in India: A cross-sectional analysis of case reports, agrometeorological and socioeconomic factors

**Journal:** Scientific Reports

**Authors:** Shivvrat Jha^1,2,3^, Manaswini Mittal^3^, Laxmi R. Prasad^4^, Somasish Ghosh Dastidar^1,3,5^, Sahana Shetty^3,6^, Damodhara Rao Mailapalli^7,8^, Pooja Kumari^9^, Harpreet Kaur^9^, Ranita Ghosh Dastidar^1,3,10#^, Chiranjay Mukhopadhyay ^1,2,3,11#^, Piyush Behari Lal^1,2,3#^

^1^Center for Emerging and Tropical Diseases, Kasturba Medical College, Manipal, Manipal Academy of Higher Education, Manipal, India

^2^Department of Microbiology, Kasturba Medical College, Manipal, Manipal Academy of Higher Education, Manipal, India

^3^Kasturba Medical College, Manipal, Manipal Academy of Higher Education, Manipal, India

^4^Department of Agricultural and Biosystems Engineering, North Dakota State University, Fargo, US

^5^Centre of Molecular Neurosciences, Kasturba Medical College, Manipal, Manipal Academy of Higher Education, Manipal, India

^6^Department of Endocrinology, Kasturba Medical College, Manipal, Manipal Academy of Higher Education, Manipal, India

^7^Agricultural and Engineering Department, Indian Institute of Technology, Kharagpur, India

^8^Agricultural and Food Engineering Department, Indian Institute of Technology, Kharagpur, India

^9^Division of Communicable Diseases, Indian Council of Medical Research, New Delhi, India

^10^Department of Biochemistry, Kasturba Medical College, Manipal, Manipal Academy of Higher Education, Manipal, India

^11^Manipal Institute of Virology, Manipal Academy of Higher Education, Manipal, India

^#^Corresponding author

Address of correspondence: piyush.lal@manipal.edu; chiranjay.m@manipal.edu; [ranita.gd@manipal.edu](mailto:ranita.gd@manipal.edu)

**Legends of all supplementary figures and tables:**

Supplementary file 1: Figure S1 to S3, pdf file (.pdf). Three supplementary figures (Figure S1 to Figure S3) with legends. Figure S1 shows spatial map for categorization of Indian States and UTs into four groups. The map was prepared using ArcGIS Desktop 10.8 software. Figure S2 is a bar graph plot for the predicted value of the diabetic population and total population in Indian States and UTs. Figure S3 shows average annual precipitation (2021) of coastal states and UTs of India.

Supplementary file 2: Table S1, excel spreadsheet (.xlsx). Supplementary table 1; Table S1 is a compilation of primary information of year-wise reported melioidosis patients and case reporting centres.

Supplementary file 3: Table S2, excel spreadsheet (.xlsx). Supplementary table 2; Table S2: A compilation of associated comorbidities reported in melioidosis patients (1953-2023).

Supplementary file 4: Table S3, excel spreadsheet (.xlsx). Supplementary table 3; Table S3 shows state wise annual average rainfall data of 30 years from 1993 to 2022. Data for the case reports from Table S1 were analyzed for different states. States were grouped into three categories based on the high, medium and low level of precipitation (Column 1st). States of northeast India and states with coastlines received high rainfall; states of central and northern India received medium or low rainfall.

Supplementary file 5: Table S4, pdf file (.pdf). Supplementary table 4; Table S4: Annual average rainfall data (year 2021) of coastal and inland areas of states and UTs that share coastline. Western ghats regions in Karnataka and coastal cities of Maharashtra had four times higher precipitation in 2021 than inland areas. Inland and coastal Odisha, Tamil Nadu and Andhra Pradesh get similar to the coastal areas possibly due to the weakened north east monsoon.

Supplementary file 6: Table S5, excel spreadsheet (.xlsx). Supplementary Table 5; Table S5 has data for area of land for rice fields in Indian states and UTs as per Government data (2016). Based on the average paddy growing area, states and union territories were grouped into three categories; States with > 3 M hectares paddy land, States with 1 to 3 M hectares paddy land and States with <1 M hectares paddy land. This data was analyzed against the number of cases mentioned in table S1.

Supplementary file 7: Table S6, excel spreadsheet (.xlsx). Supplementary Table 6; Table S6 has population matrix of Indian States and UTs. Table consists of the total, rural and urban population (Government Census Data 2011) along with the total number of Indian melioidosis cases (1953-2023; Table S1), cases per million total population and per million rural population.

Supplementary file 8: Table S7, excel spreadsheet (.xlsx). Supplementary Table 7; Table S7 contains data for diabetic prevalence in Indian States and UTs. Number of DM patients in each state was calculated based on the percentage of diabetic people in each state mentioned in NCD clinics database (2021). This data was compared with the total number of Indian melioidosis cases (1953-2023; table S1). States are grouped based on the number of diabetic patients in each state.

Supplementary file 9: Table S8, excel spreadsheet (.xlsx). Supplementary Table 8; Table S8 has data for diagnostic centres for melioidosis in India. The table consists of the total number of centres/hospitals/institutes from where cases have been reported (1953-2023) along with the total number of Indian melioidosis cases (1953-2023; table S1).

Supplementary file 10: Table S9, excel spreadsheet (.xlsx). Supplementary Table 9; Table S9 contains soil properties of Indian states and UTs associated with growth of *B. pseudomallei* along with the total number of Indian melioidosis cases (1953-2023; table S1). Ten properties has been considered for optimum growth of *B. pseudomallei* a) non saline (0-1.68dS/m) b)  nitrogen- Low (<280kg/ha) c) organic carbon- Low (<0.50%) d) iron- sufficient (>4.5ppm) e) manganese- sufficient (>2ppm) f) phosphorus- high (>25 kg/ha) g) potassium- high (>280kg/ha) h) zinc- sufficient (>0.6ppm) i) copper- sufficient (>0.2ppm) j) normal pH (>6.5-7.5), strongly acidic (<4.5), moderately acidic (4.5-5.5), slightly acidic, (>5.5-6.5) and slightly alkaline (>7.5-8.5). In this excel file (ten worksheets), we have collected soil mineral content for each state and compared the values against number of melioidosis cases.

Supplementary file 11: Table S10, excel spreadsheet (.xlsx). Supplementary Table 10; Table S10 has data for state and UT wise maximum temperature (April) along with the total number of Indian melioidosis cases (1953-2023; table S1). All states and UTs were categorized based on the highest mean temperature in April and compared the data with the number of case reports. Six states, Bihar, Andhra Pradesh, Chhattisgarh, Maharashtra, Odisha and Telangana, had the highest mean temperature above 35°C. 16 states had the highest mean temperature in the range of 30°C to 34°C. 14 states had the highest mean temperature in the range of 22°C to 30°C.

Supplementary file 12: Table S11, excel spreadsheet (.xlsx). Supplementary Table 11; Table S11 has three sheets (Table 11 a, Table 11 b, Table 11 c) containing data for melioidosis cases from the Udupi reported at Kasturba Hospital, Manipal and precipitation data in Udupi during 2010 to 2023. There are three IMD-Pune rain gauge stations in Udupi district. We collected average of precipitation of all three stations for the duration of 2010 - 2022. We gathered case report information of patients of this region for the same duration and compared the data. Table a) has month-wise number of melioidosis cases with age and gender. * denotes missing of data point. Table b) has Annual number of melioidosis cases with age and gender and annual rainfall (mm) data of Udupi. Table c has Month-wise number of melioidosis cases with month-wise average rainfall (mm) data of Udupi.

Supplementary file 13: Table S12, pdf file (.pdf). Supplementary Table 12; Table S12 shows migration chart of patients across different states for diagnosis of melioidosis in southern part of India. Data were derived from information mentioned in table S1.

Supplementary file 14: Table S13, excel spreadsheet (.xlsx). Supplementary Table 13; Table S13 has two sheets containing Melioidosis checklist index and Melioidosis probability factors. Table S13 a) shows the "Melioidosis checklist index" for the melioidosis case reporting. Table S13 b) shows Melioidosis probability factors in Indian states and UTs based on the analysis of case reports and other factors studied in this work.
