# Supplementary material for: Missing links of melioidosis in India: a cross-sectional analysis of case reports, agrometeorological and socioeconomic factors
Source: Sci Rep. 2025 Nov 29;15:43237. doi: 10.1038/s41598-025-27178-4 (PMC12680645; doi:10.1038/s41598-025-27178-4)
Supplement: Supplementary file 2 — Supplementary Material 2 [file 41598_2025_27178_MOESM2_ESM.docx]

**Title:** Missing links of melioidosis in India: A cross-sectional analysis of case reports, agrometeorological and socioeconomic factors

**Journal:** Scientific Reports

**Authors:** Shivvrat Jha^1,2,3^, Manaswini Mittal^3^, Laxmi R. Prasad^4^, Somasish Ghosh Dastidar^1,3,5^, Sahana Shetty^3,6^, Damodhara Rao Mailapalli^7,8^, Pooja Kumari^9^, Harpreet Kaur^9^, Ranita Ghosh Dastidar^1,3,10#^, Chiranjay Mukhopadhyay ^1,2,3,11#^, Piyush Behari Lal^1,2,3#^

^1^Center for Emerging and Tropical Diseases, Kasturba Medical College, Manipal, Manipal Academy of Higher Education, Manipal, India

^2^Department of Microbiology, Kasturba Medical College, Manipal, Manipal Academy of Higher Education, Manipal, India

^3^Kasturba Medical College, Manipal, Manipal Academy of Higher Education, Manipal, India

^4^Department of Agricultural and Biosystems Engineering, North Dakota State University, Fargo, US

^5^Centre of Molecular Neurosciences, Kasturba Medical College, Manipal, Manipal Academy of Higher Education, Manipal, India

^6^Department of Endocrinology, Kasturba Medical College, Manipal, Manipal Academy of Higher Education, Manipal, India

^7^Agricultural and Engineering Department, Indian Institute of Technology, Kharagpur, India

^8^Agricultural and Food Engineering Department, Indian Institute of Technology, Kharagpur, India

^9^Division of Communicable Diseases, Indian Council of Medical Research, New Delhi, India

^10^Department of Biochemistry, Kasturba Medical College, Manipal, Manipal Academy of Higher Education, Manipal, India

^11^Manipal Institute of Virology, Manipal Academy of Higher Education, Manipal, India

^#^Corresponding author

Address of correspondence: piyush.lal@manipal.edu; chiranjay.m@manipal.edu; [ranita.gd@manipal.edu](mailto:ranita.gd@manipal.edu)

**Supplementary figures (Figure S1 to Figure S3) with legends.**


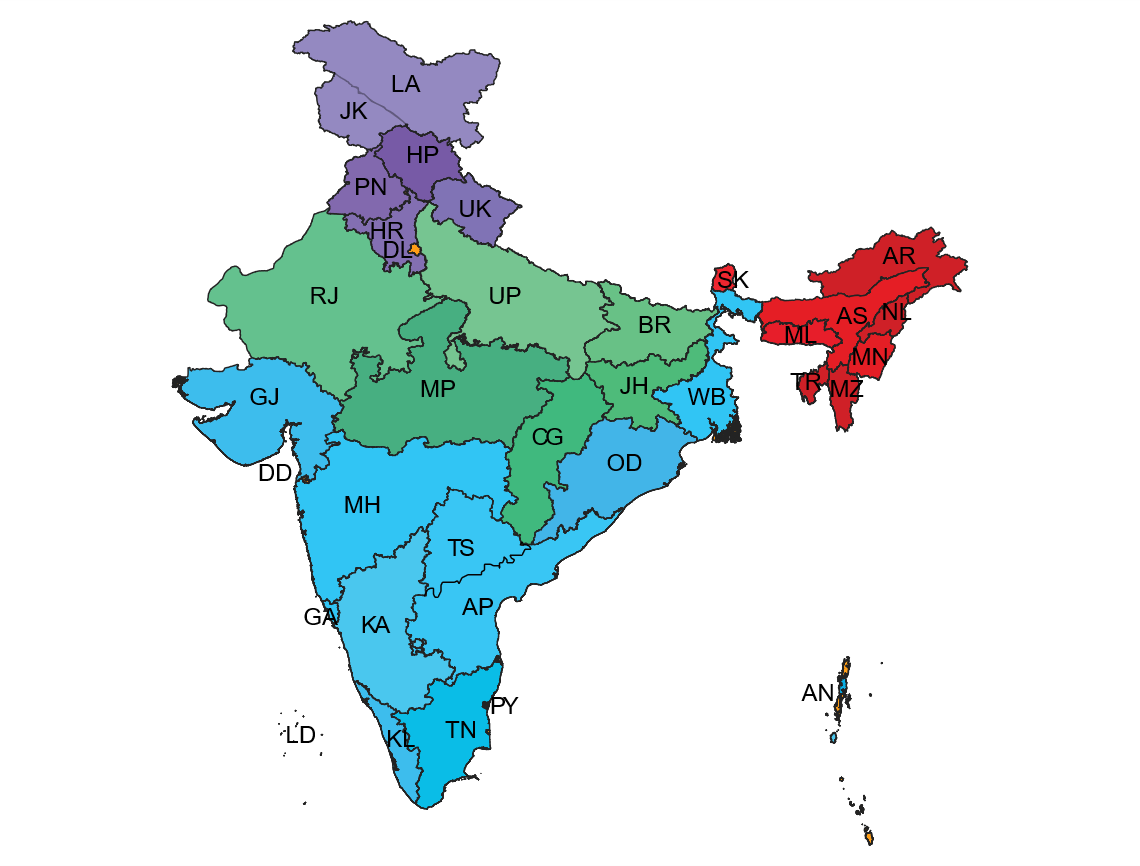


**Figure S1: Spatial map for categorization of Indian States and UTs into four groups**. States and UTs with coastline were marked with blue color, Inland northeast states were marked with red color, inland central states including Rajasthan were marked with green color, and inland northern states and UTs were marked with purple color. Regardless of geography/region (east, west, north, and south), all states and Union Territories (UTs) with sea/ocean coast were grouped into states with coastline category. Although Rajasthan state falls in the western non-coastline category because of its climate similarity to that of central Indian states, it is grouped into the central states without coastline category. The map was prepared based on recent government data using ArcGIS Desktop 10.8 software. State and Union Territories (UTs) were mentioned as code letters and used the same codes throughout the study. Andaman and Nicobar- AN, Andhra Pradesh- AP, Arunachal Pradesh- AR, Assam-AS, Bihar-BR, Chandigarh- CH, Dadra and Nagar Haveli and Daman and Diu- DD, Delhi- DL, Goa- GA, Gujarat- GJ, Haryana- HR, Himachal Pradesh- HP, Jammu and Kashmir- JK, Karnataka- KA, Kerala- KL, Lakshadweep- LD, Madhya Pradesh- MP, Maharashtra- MH, Manipur- MN, Meghalaya- ML, Mizoram- MZ, Nagaland- NL, Odisha- OD, Puducherry- PY, Punjab- PN, Rajasthan- RJ, Sikkim- SK, Tamil Nadu- TN, Tripura- TR, Uttar Pradesh- UP and West Bengal- WB. The map was prepared using ArcGIS Desktop 10.8 software.


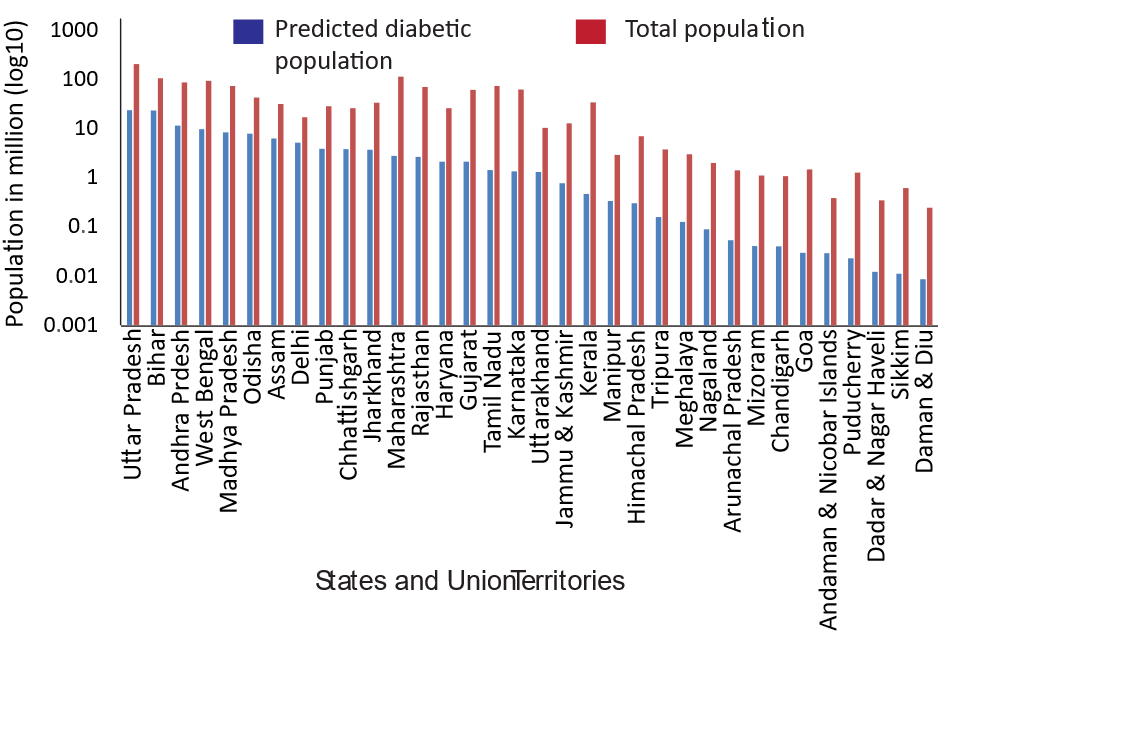


**Figure S2: A bar graph plot for the predicted value of the diabetic population and total population in Indian States and UTs**. The red bars represent the total population (2011 census), and the blue bars represent the predicted number of diabetic populations.


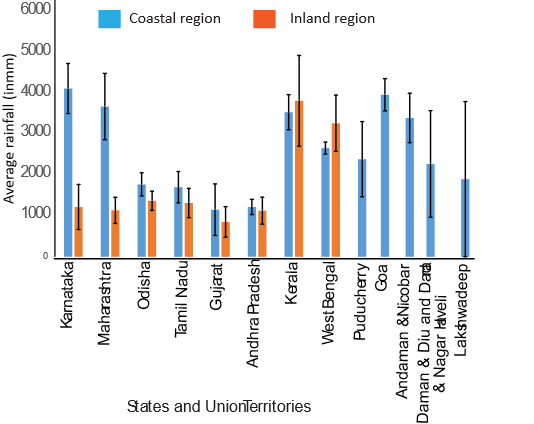


**Figure S3: Average annual precipitation (2021) of coastal states and UTs of India.** The blue bars represent average annual precipitation of coastal regions and the orange bars represent average the annual precipitation of inland regions of the same state. The precipitation data is the average of 12 months of rainfall data in 2021 for the stations that belong to the coastal or inland regions of the states. Data were obtained from IMD Pune for each station of the states and UTs. The error bars are for the standard deviation of the precipitation level of all rainfall stations of the coastal or inland regions.
