# Supplementary material for: Missing links of melioidosis in India: a cross-sectional analysis of case reports, agrometeorological and socioeconomic factors
Source: Sci Rep. 2025 Nov 29;15:43237. doi: 10.1038/s41598-025-27178-4 (PMC12680645; doi:10.1038/s41598-025-27178-4)
Supplement: Supplementary file 6 — Supplementary Material 6 [file 41598_2025_27178_MOESM6_ESM.docx]

**Title:** Missing links of melioidosis in India: A cross-sectional analysis of case reports, agrometeorological and socioeconomic factors

**Journal:** Scientific Reports

**Authors:** Shivvrat Jha^1,2,3^, Manaswini Mittal^3^, Laxmi R. Prasad^4^, Somasish Ghosh Dastidar^1,3,5^, Sahana Shetty^3,6^, Damodhara Rao Mailapalli^7,8^, Pooja Kumari^9^, Harpreet Kaur^9^, Ranita Ghosh Dastidar^1,3,10#^, Chiranjay Mukhopadhyay ^1,2,3,11#^, Piyush Behari Lal^1,2,3#^

^1^Center for Emerging and Tropical Diseases, Kasturba Medical College, Manipal, Manipal Academy of Higher Education, Manipal, India

^2^Department of Microbiology, Kasturba Medical College, Manipal, Manipal Academy of Higher Education, Manipal, India

^3^Kasturba Medical College, Manipal, Manipal Academy of Higher Education, Manipal, India

^4^Department of Agricultural and Biosystems Engineering, North Dakota State University, Fargo, US

^5^Centre of Molecular Neurosciences, Kasturba Medical College, Manipal, Manipal Academy of Higher Education, Manipal, India

^6^Department of Endocrinology, Kasturba Medical College, Manipal, Manipal Academy of Higher Education, Manipal, India

^7^Agricultural and Engineering Department, Indian Institute of Technology, Kharagpur, India

^8^Agricultural and Food Engineering Department, Indian Institute of Technology, Kharagpur, India

^9^Division of Communicable Diseases, Indian Council of Medical Research, New Delhi, India

^10^Department of Biochemistry, Kasturba Medical College, Manipal, Manipal Academy of Higher Education, Manipal, India

^11^Manipal Institute of Virology, Manipal Academy of Higher Education, Manipal, India

^#^Corresponding author

Address of correspondence: piyush.lal@manipal.edu; chiranjay.m@manipal.edu; [ranita.gd@manipal.edu](mailto:ranita.gd@manipal.edu)

**Table S4: Annual average rainfall data (year 2021) of coastal and inland areas of states and UTs that share coastline.** Western ghats regions in Karnataka and coastal cities of Maharashtra had four times higher precipitation in 2021 than inland areas. Inland and coastal Odisha, Tamil Nadu and Andhra Pradesh get similar to the coastal areas possibly due to the weakened north east monsoon.

|  | **Average rainfall** | | **Standard deviation of average rainfall** | |
| --- | --- | --- | --- | --- |
| **States/UTs** | **Coastal regions** | **Inland regions** | **Coastal regions** | **Inland regions** |
| Karnataka | 4115.9 | 1219.5 | 612.352 | 550.9141 |
| Maharashtra | 3674.6 | 1136.5 | 811.371 | 320.2635 |
| Odisha | 1770.4 | 1370.5 | 282.137 | 235.8141 |
| TamilNadu Coastal | 1703.3 | 1315 | 383.771 | 358.2877 |
| Gujarat | 1151.6 | 854.8 | 630.9 | 372.5 |
| Andhra Pradesh | 1219.6 | 1129.6 | 186.405 | 330.0489 |
| Kerala | 3537.4 | 3817 | 430.166 | 1109.38 |
| West BENGAL | 2659.6 | 3268.3 | 147.149 | 687.0706 |
| Puducherry | 2386.9 | 0 | 920.783 | 0 |
| Goa | 3964.6 | 0 | 395.273 | 0 |
| Andaman & Nicobar | 3396.7 | 0 | 605.031 | 0 |
| Daman Diu & Dadra Nagar Haveli | 2272.4 | 0 | 1303.95 | 0 |
| Lakshadweep | 1898.7 | 0 | 1898.7 | 0 |
