# Supplementary material for: Missing links of melioidosis in India: a cross-sectional analysis of case reports, agrometeorological and socioeconomic factors
Source: Sci Rep. 2025 Nov 29;15:43237. doi: 10.1038/s41598-025-27178-4 (PMC12680645; doi:10.1038/s41598-025-27178-4)
Supplement: Supplementary file 14 — Supplementary Material 14 [file 41598_2025_27178_MOESM14_ESM.docx]

**Title:** Missing links of melioidosis in India: A cross-sectional analysis of case reports, agrometeorological and socioeconomic factors

**Journal:** Scientific Reports

**Authors:** Shivvrat Jha^1,2,3^, Manaswini Mittal^3^, Laxmi R. Prasad^4^, Somasish Ghosh Dastidar^1,3,5^, Sahana Shetty^3,6^, Damodhara Rao Mailapalli^7,8^, Pooja Kumari^9^, Harpreet Kaur^9^, Ranita Ghosh Dastidar^1,3,10#^, Chiranjay Mukhopadhyay ^1,2,3,11#^, Piyush Behari Lal^1,2,3#^

^1^Center for Emerging and Tropical Diseases, Kasturba Medical College, Manipal, Manipal Academy of Higher Education, Manipal, India

^2^Department of Microbiology, Kasturba Medical College, Manipal, Manipal Academy of Higher Education, Manipal, India

^3^Kasturba Medical College, Manipal, Manipal Academy of Higher Education, Manipal, India

^4^Department of Agricultural and Biosystems Engineering, North Dakota State University, Fargo, US

^5^Centre of Molecular Neurosciences, Kasturba Medical College, Manipal, Manipal Academy of Higher Education, Manipal, India

^6^Department of Endocrinology, Kasturba Medical College, Manipal, Manipal Academy of Higher Education, Manipal, India

^7^Agricultural and Engineering Department, Indian Institute of Technology, Kharagpur, India

^8^Agricultural and Food Engineering Department, Indian Institute of Technology, Kharagpur, India

^9^Division of Communicable Diseases, Indian Council of Medical Research, New Delhi, India

^10^Department of Biochemistry, Kasturba Medical College, Manipal, Manipal Academy of Higher Education, Manipal, India

^11^Manipal Institute of Virology, Manipal Academy of Higher Education, Manipal, India

^#^Corresponding author

Address of correspondence: piyush.lal@manipal.edu; chiranjay.m@manipal.edu; [ranita.gd@manipal.edu](mailto:ranita.gd@manipal.edu)

**Table S12: Migration chart of patients across different states for diagnosis of melioidosis in southern part of India.** Data were derived from information mentioned in Table S1.

| State where the disease was diagnosed | | State where patients belonged to | |
| --- | --- | --- | --- |
| Name of the state | Diagnostic center | Native state of the patient | No. of patients |
| Odisha | AIIMS,  Bhuvneshwar | West Bengal | 4 |
|  |  | UP | 2 |
| Tamil Nadu | Apollo Hospital, Chennai | Assam | 3 |
|  |  | Andaman and Nicobar | 1 |
|  | Christian Medical College (CMC), Vellore | Arunachal Pradesh | 1 |
|  |  | Assam | 13 |
|  |  | Bihar | 2 |
|  |  | Chhattisgarh | 4 |
|  |  | Gujarat | 1 |
|  |  | Jharkhand | 33 |
|  |  | Madhya Pradesh | 1 |
|  |  | North east India | 5 |
|  |  | Odisha | 8 |
|  |  | Tripura | 5 |
|  |  | Uttar Pradesh | 1 |
|  |  | West Bengal | 49 |
